# Mechanistic insights into interfacial failure of hard carbon anodes in sodium-ion batteries under extreme conditions

**DOI:** 10.1039/d6sc03211g

**Published:** 2026-05-21

**Authors:** Rongfen Feng, Qiang Wang, Xiaosha Wu, Hanqi Zhang, Yang Gao, Ning Zhao, Jiahe Li, Xiuxia Zhao, Xiaofei Hu

**Affiliations:** a School of Chemistry, Xi'an Jiaotong University Xi'an 710049 Shaanxi China; b National Innovation Platform (Center) for Industry-Education Integration of Energy Storage Technology, Xi'an Jiaotong University Xi'an 710049 Shaanxi China xiaofei.hu@xjtu.edu.cn; c CATARC Intelligent Technology (Tianjin) Co., Ltd Tianjin 300300 China

## Abstract

Revealing the failure mechanisms of hard carbon (HC) anodes under extreme temperatures is crucial for the wide-temperature applications of sodium-ion batteries (SIBs). *Via* electrochemical tests and multi-scale characterization, we systematically studied the dominant failure mechanisms of HC anodes at −20 °C and 60 °C. At −20 °C, low temperature reduces electrolyte ionic conductivity and increases the interfacial charge transfer barrier. This causes overall kinetic sluggishness, which further induces Na dendrite growth and continuous solid electrolyte interphase (SEI) thickening. Both processes accelerate active sodium consumption and interfacial impedance accumulation, ultimately leading to irreversible capacity decay. Notably, we observed for the first time a crystalline SEI layer rich in nanograins on the HC surface after high-temperature cycling at 60 °C, which differs from the conventional view that high temperatures simply cause disordered SEI thickening. Although this crystallization suppresses excessive SEI growth, its dense lattice structure significantly raises the Na^+^ desolvation energy barrier and interfacial migration impedance. The resulting high overpotential further drives electrolyte decomposition, forming a vicious cycle of: “aggravated side reactions → SEI crystallization → increased impedance → further side reactions”. More critically, HC anode degradation is dominated by interfacial failure rather than bulk damage, providing a theoretical reference for wide-temperature SIB design and optimization.

## Abstract 2

Hard carbon degradation pathways at extreme temperatures are revealed: at −20 °C, sluggish kinetics cause dendrite growth and continuous SEI thickening; at 60 °C, the SEI nanocrystallization raises Na^+^ desolvation barrier and interfacial impedance.

## Graphical abstract

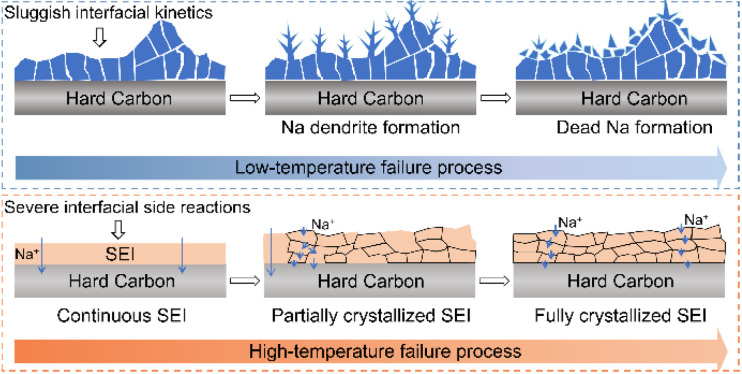

## Introduction

With the rapid popularization of electric vehicles and the fast development of energy storage systems, the reliability of batteries in real-world application scenarios—particularly under extreme high and low temperature conditions such as polar regions, high-altitude areas and deserts—has become a critical bottleneck restricting their large-scale deployment.^1^ Among various emerging electrochemical energy storage technologies, sodium-ion batteries (SIBs) have emerged as a promising supplement and even potential alternative to lithium-ion batteries (LIBs), owing to their abundant sodium raw materials, high cost-effectiveness and superior intrinsic safety. However, compared with the relatively mature LIB technology, SIBs still face severe challenges in practical wide-temperature applications: extreme operating conditions (ultra-low/high temperature) can significantly exacerbate internal side reactions, and trigger unstable solid electrolyte interphase (SEI) formation, sodium dendrite growth and localized overheating, which in turn lead to rapid performance degradation and even serious safety incidents such as thermal runaway.^2,3^ Clarifying the failure mechanisms of SIBs under extreme temperatures is thus of crucial theoretical and practical significance for breaking through their wide-temperature application bottlenecks.

Current research on SIB degradation has confirmed that the anode serves as a key regulatory unit for the overall aging behavior of full cells, with its failure mainly involving SEI aging, active material loss, sodium dendrite growth and electrode structural degradation.^4^ Existing studies have preliminarily revealed that elevated temperatures will aggravate the electrode/electrolyte interfacial side reactions of SIBs, while low temperatures will induce sluggish Na^+^ diffusion kinetics and severe electrode polarization.^5^ Notably, due to the higher chemical reactivity of sodium than lithium, the interfacial reactions of SIBs are inherently more severe than those of LIBs, and their failure mechanisms under extreme temperatures exhibit more complex temperature dependence.^6^ However, the current research on SIB extreme temperature failure remains fragmented: most studies only focus on a single temperature condition or a single failure phenomenon, and lack systematic comparative research on the intrinsic differences of failure mechanisms under extreme low and high temperatures, as well as the in-depth excavation of the internal correlation between reaction kinetics, interfacial structure evolution and electrode bulk performance degradation. To date, the dominant failure pathways of the core hard carbon (HC) anode of SIBs under extreme temperatures, the evolutionary law of SEI structure and its regulatory mechanism on Na^+^ transport, and the intrinsic link between interfacial failure and bulk structural changes of HC have not been clearly elucidated, which severely restricts the targeted design of wide-temperature-adaptable SIB electrode materials and electrolyte systems.

In this work, we conduct a systematic and comparative study on the failure mechanisms of HC anodes under extreme low temperature (−20 °C) and high temperature (60 °C) by combining a series of electrochemical characterization techniques and advanced material characterization methods (*e.g.*, high-resolution transmission electron microscopy (HRTEM), X-ray photoelectron spectroscopy (XPS)). We clarify the differentiated dominant failure pathways of HC anodes under two extreme temperature conditions, reveal the evolution characteristics of SEI structure and composition at the atomic scale, and establish the correlation among interfacial kinetic changes, SEI structural degradation, sodium dendrite growth and HC bulk structural damage. This study constructs a complete failure mechanism system of HC anodes under extreme high and low temperatures, and identifies the core limiting factors of SIB performance under different extreme temperature conditions, providing an important theoretical basis and targeted guidance for the optimized design of high-performance SIBs with a wide temperature window.

## Results and discussion

**Fig. 1:**
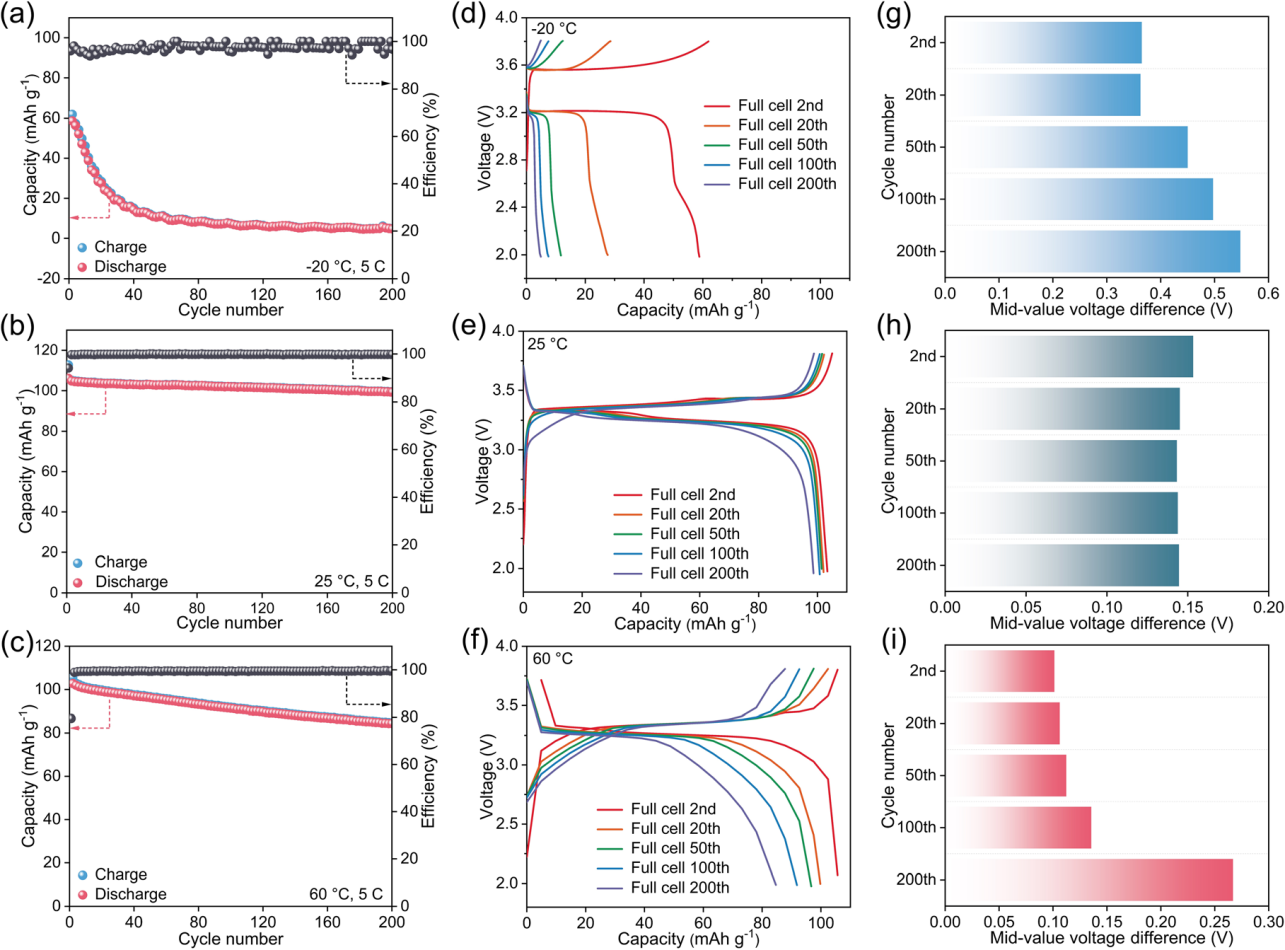
Cycling performance of NVP//HC batteries between 2.0 and 3.8 V tested at (a) −20 °C, (b) 25 °C and (c) 60 °C. Charge and discharge curves of NVP//HC batteries between 2.0 and 3.8 V tested at (d) −20 °C, (e) 25 °C and (f) 60 °C. Mid-value voltage difference for charge and discharge processes of NVP//HC batteries between 2.0 and 3.8 V tested at (g) −20 °C, (h) 25 °C and (i) 60 °C.

Fig. 1a–c illustrate the long-term performance of the Na_3_V_2_(PO_4_)_3_ (NVP)//HC full cell at a rate of 5C (1C = 117 mA g^−1^) under different temperature conditions of −20 °C, 25 °C, and 60 °C. At 25 °C and 60 °C, the cell capacity exhibits an approximately linear decay trend, yet the decay rates differ remarkably. Specifically, at 25 °C, the cell delivers an initial capacity of 104.81 mAh g^−1^ and still retains 99.28 mAh g^−1^ after 200 cycles, corresponding to a capacity retention rate of 94.72% with an average per-cycle capacity loss of merely 0.0277 mAh g^−1^. In contrast, at 60 °C, the capacity retention rate drops to 81.95% after 200 cycles, with an average per-cycle capacity fading of 0.0927 mAh g^−1^, which is 3.35 times that at 25 °C. This result demonstrates that elevated temperature can drastically accelerate the cell aging process. In stark contrast, the cell performance under the low-temperature condition of −20 °C presents completely different decay characteristics: its initial discharge capacity plummets to 58.67 mAh g^−1^, accompanied by a rapid capacity fading trend during subsequent cycling. This phenomenon reflects the severe suppression effect of low temperatures on the kinetic properties and reversible capacity of the cell. In terms of initial coulombic efficiency (ICE), at 25 °C, the cell delivers a high ICE of 94.11%, while the ICE slightly increases to 96.04% at −20 °C due to suppressed reaction kinetics that reduce the initial charge capacity. In contrast, the ICE drops sharply to 79.45% at 60 °C, as aggravated reductive decomposition of the electrolyte on the anode consumes active sodium ions and electrons and further impairs discharge capacity. Collectively, these results indicate that both extreme high and low temperatures can expedite the degradation of cell performance, but the decay kinetics and dominant mechanisms underlying these two scenarios are inherently distinct. Therefore, systematic elucidation of the capacity fading mechanisms under different extreme temperatures provides crucial guidance for the development of SIBs adaptable to a wide temperature range.

Fig. 1d–f depict the evolution of typical charge–discharge profiles of the NVP//HC full-cell with increasing cycle numbers at −20 °C, 25 °C and 60 °C, respectively. Across all temperature cases, it is observed that the polarization degree of charge–discharge profiles gradually intensifies with prolonged cycling, characterized by an increase in the charge plateau voltage, a decrease in the discharge plateau voltage, and a continuous expansion of the voltage gap between them. This phenomenon reflects the cumulative internal resistance and the degradation of kinetic performance of the cell during cycling. In terms of polarization degree, the overpotential at −20 °C is the most pronounced: a large charge–discharge voltage gap is already observed in the initial cycle (with a median voltage difference of 0.3649 V), and the polarization level deteriorates rapidly with cycling. By the 200th cycle, the median voltage difference surges to 0.5477 V. In contrast, the cell exhibits significantly lower polarization at 25 °C, with a median voltage difference of only 0.1533 V in the 2nd cycle. Moreover, the polarization increases extremely slowly during cycling, and the median voltage difference remains stable at 0.1455 V even after 200 cycles, indicating excellent interfacial stability and cycling reversibility of the cell at this temperature. The polarization behavior at 60 °C shows certain complexity: in the early stage of cycling, benefiting from the enhanced ionic conductivity, the overpotential is slightly lower than that at 25 °C (with a median voltage difference of 0.1012 V in the 2nd cycle). Nevertheless, the polarization growth rate accelerates markedly with the progression of cycling, and the median voltage difference rises to 0.2608 V by the 200th cycle, exceeding the corresponding value at 25 °C. This result reveals that while high temperatures improve the ion transport kinetics, they also accelerate the occurrence of irreversible processes such as electrolyte decomposition and interfacial side reactions, thereby leading to a faster upward trend of polarization degree during cycling.

**Fig. 2:**
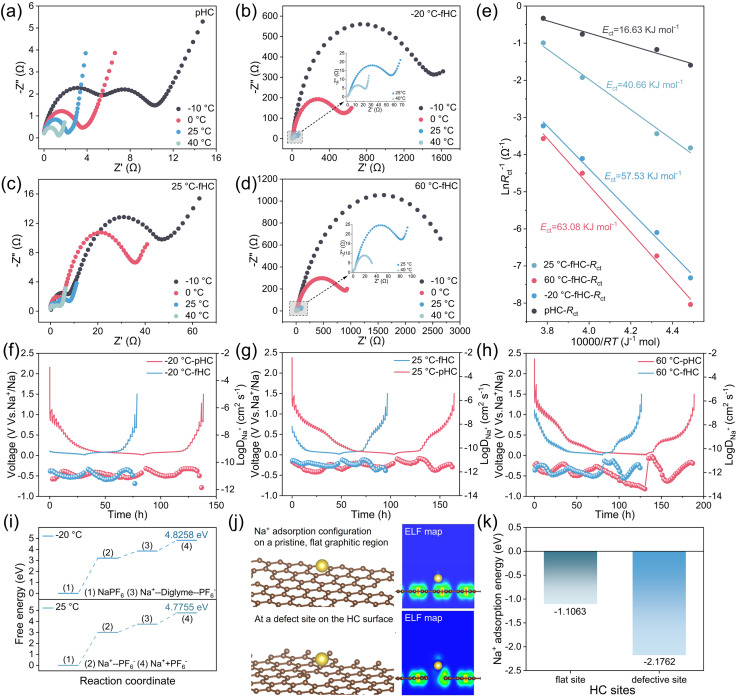
Nyquist plots of (a) pHC//Na batteries, (b) −20 °C-fHC//Na batteries, (c) 25 °C-fHC//Na batteries and (d) 60 °C-fHC//Na batteries at different temperatures and (e) corresponding fitted Arrhenius plots. GITT curves and the calculated *D*_Na^+^_ of pHC//Na and fHC//Na at (f) −20 °C, (g) 25 °C and (h) 60 °C. (i) The dissociation free energy of NaPF_6_ salt in a diglyme solvent matrix at −20 °C and 25 °C. (j) Theoretical calculations of Na^+^ adsorption configurations and corresponding ELF maps on HC surfaces. (k) Adsorption energy of Na^+^ on flat and defect HC sites.

Fig. 2 comprehensively presents the electrochemical impedance spectroscopy (EIS) and galvanostatic intermittent titration technique (GITT) curves of HC (including pristine HC (pHC) electrodes and post-cycling-failure HC (fHC) electrodes) half-cells, systematically revealing the charge transfer and ion diffusion kinetic behaviors of pHC and fHC anodes at different temperatures. Fig. 2a–d display the Nyquist plots of half-cells assembled with pHC and fHC (after undergoing cycling at −20 °C, 25 °C, and 60 °C) tested under various temperatures, respectively. These characteristic features correspond sequentially to the impedance of the SEI (*R*_SEI_), charge-transfer impedance (*R*_ct_), and the sodium-ion diffusion process within the electrode bulk.^7^ By comparison, compared with pHC electrodes, the fHC electrodes cycled at any temperature exhibit a significantly increased radius of the mid-frequency capacitive arc. This observation confirms that the cycling process universally induces an increase in charge transfer impedance. Notably, the −20 °C-fHC and 60 °C-fHC electrodes show the most pronounced impedance growth under all tested temperatures. Particularly under low-temperature test conditions, the capacitive arc expands sharply, demonstrating that low- and high-temperature cycling-induced failure causes severe damage to the electrode/electrolyte interface. Fig. 2e shows the Arrhenius plots fitted from the temperature-dependent EIS data. The desolvation activation energies of sodium ions at the electrolyte/pHC and electrolyte/fHC interfaces were calculated by fitting the slopes of the Arrhenius plots and subsequently compared.^8^ The results reveal that the desolvation activation energies of all fHC electrodes are higher than those of the pHC electrode, indicating that the failure process elevates the energy barrier of the electrode reaction.^9^ Among these, the 60 °C-fHC exhibits the highest desolvation activation energy, which is consistent with its maximum impedance increment. This quantitatively verifies from a kinetic perspective that high-temperature cycling induces the most severe interfacial degradation, thereby exacerbating the difficulty of the subsequent charge transfer process. Fig. 2f–h display the GITT curves and the calculated sodium-ion chemical diffusion coefficients (*D*_Na^+^_) of pHC half-cells and their corresponding temperature-cycled fHC half-cells tested at −20 °C, 25 °C, and 60 °C, respectively. Overall, at all tested temperatures, despite the significant increase in interfacial impedance of HC after cycling-induced failure (evidenced by the capacitive semicircles in Fig. 2a–d), the bulk *D*_Na^+^_ of HC remains essentially unchanged. This comparative result reveals the core of the HC failure mechanism: its primary degradation originates from the electrode/electrolyte interface rather than the bulk interior of the material.

To quantitatively understand the limitations of the electrolyte at sub-ambient temperatures, we calculated the dissociation free energy of NaPF_6_ within a diglyme solvent matrix (Fig. 2i). At 25 °C, the dissociation free energy was calculated to be 4.7755 eV. Upon decreasing the temperature to −20 °C, this value increased to 4.8258 eV. This increase in dissociation free energy quantitatively rationalizes the deteriorated ionic conductivity observed at low temperatures. A higher thermodynamic barrier indicates that the separation of Na^+^ from PF_6_^−^ becomes less favorable under cold conditions, thereby reducing the concentration of free charge carriers and lowering the overall ionic conductivity.^10,11^

The uniformity of Na deposition on HC anodes strongly depends on the surface affinity toward Na^+^. To clarify the microscopic origin of inhomogeneous Na plating—a critical precursor to dendrite formation (Fig. 2j), we calculated the Na^+^ adsorption energy on two representative HC surface models: a pristine flat basal plane and a defective surface site. The calculations reveal a clear energetic preference for Na^+^ adsorption at defect sites (Fig. 2k). The adsorption energy at the defective site reaches −2.1762 eV, nearly twice as favorable as that on the flat basal plane (−1.1063 eV). This distinct difference in adsorption energetics provides a straightforward microscopic explanation for experimentally observed inhomogeneous Na deposition, especially at low temperatures with restricted ion mobility. Owing to their deeper energy wells, defect sites serve as preferential nucleation centers for Na^+^. During plating, Na^+^ tends to reduce and deposit dominantly at thermodynamically favorable defects rather than on flat regions. Such biased nucleation induces localized accumulation and initial protrusions.^12^ These protrusions further concentrate the local electric field, accelerating subsequent Na^+^ reduction and facilitating dendrite evolution. Accordingly, the computed difference in adsorption energies confirms that intrinsic surface defects dominate inhomogeneous Na nucleation, eventually triggering dendrite growth and accompanying safety risks.^13,14^

**Fig. 3:**
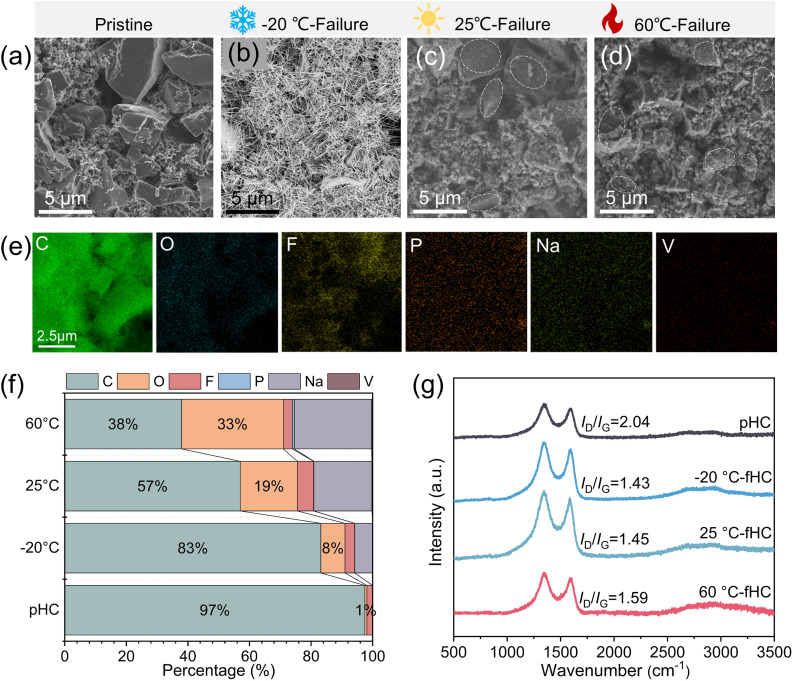
(a–d) SEM images of pHC, −20 °C-fHC, 25 °C-fHC and 60 °C-fHC. (e) EDS mapping of pHC. (f) Weight percentages (%) of elements from EDS mappings for pHC, −20 °C-fHC, 25 °C-fHC and 60 °C-fHC. (g) Raman spectra of pHC, −20 °C-fHC, 25 °C-fHC and 60 °C-fHC.

To investigate the effects of failure conditions at different temperatures on the surface microstructure and elemental composition of HC, this study employed scanning electron microscopy (SEM) coupled with energy-dispersive X-ray spectroscopy (EDS) to conduct systematic characterization and comparative analysis of pHC electrode sheets as well as those subjected to failure treatments at varied temperatures.^15^ The characterization results demonstrate that the surface of the pHC particles (Fig. 3a) exhibits a relatively smooth, dense microscopic morphology with distinct particle boundaries, free from obvious defects or impurity attachments. EDS elemental analysis reveals that only a strong C signal is detected on the sample surface, accompanied by weak F and extremely faint O signals. The presence of F and O elements is primarily attributed to the polyvinylidene fluoride (PVDF) binder used during electrode fabrication, which is consistent with the inherent compositional characteristics of HC and the electrode preparation system.^16,17^ In sharp contrast, both the surface morphology and elemental composition of HC undergo significant alterations after failure treatments at different temperatures. Under −20 °C, typical dendrite-like protrusions are clearly observed on the surface of HC (Fig. 3b). The formation of such dendrite structures is hypothesized to be closely associated with the sluggish Na^+^ insertion/extraction kinetics and heterogeneous interfacial charge transfer on the HC surface in low-temperature environments. These phenomena further induce local electrochemical reaction imbalance, ultimately triggering dendrite growth. The growth of sodium dendrites not only tends to pierce the battery separator and cause internal short circuits but also forms “dead sodium”, leading to irreversible loss of active materials.^18^ Meanwhile, it is accompanied by significant volume expansion effects that damage the structural integrity of the HC bulk. Thus, sodium dendrite growth is identified as the core inducement for battery performance degradation and failure under low-temperature conditions. After failure at 25 °C, the surface roughness of HC particles is significantly higher than that of pHC (Fig. 3c); the originally regular particle edges become blurred, and a large number of fine debris-like substances adhere to the surface.^19^ This is presumed to result from slight peeling or structural disintegration of material particles during the failure process. EDS mapping analysis indicates that, compared with pHC, the atomic percentage of C on the surface of 25 °C-fHC decreases slightly, while the atomic percentage of Na increases correspondingly. This observation suggests that Na^+^ ions have undergone a certain degree of adsorption and residual accumulation on the HC surface at 25 °C, accompanied by mild interfacial reactions. The high-temperature failure condition exerts a more pronounced destructive effect on the surface structure of HC (Fig. 3d). Its surface roughness is further increased compared with that of 25 °C-fHC; the surface of 60 °C-fHC particles is covered by a distinct layer of deposits and flocculent products, and penetrating cracks can be observed on some particles, presenting a striking contrast to the smooth and dense surface morphology of pHC. In addition to C, O, F, P, and Na derived from the HC matrix and electrolyte decomposition, V signals are also detected on 25 °C-fHC and 60 °C-fHC, which originate from the dissolution of vanadium ions from the NVP cathode during cycling.^20^

In addition, to elucidate the effects of failure processes at different temperatures on the internal graphitization structure and crystal order of HC materials, Raman spectroscopy was employed in this study to characterize both pHC samples and those subjected to failure treatments under varied temperature conditions.^21^ For HC materials, the characteristic D and G bands in Raman spectra serve as core indicators for reflecting their graphitization degree, crystal order, and structural defect density. Specifically, the D band is located at approximately 1350 cm^−1^, corresponding to the disordered carbon structures, edge defects of graphite layers, and lattice distortion regions within the material. The G band appears at around 1580 cm^−1^, which originates from the in-plane vibrational modes of six-membered carbon rings composed of sp^2^-hybridized carbon atoms with high graphitization levels. The intensity ratio of these two bands (*I*_D_/*I*_G_) acts as a key quantitative metric for evaluating the graphitization degree and structural order of HC. A higher *I*_D_/*I*_G_ ratio indicates more internal defects, lower graphitization degree, and poorer structural order of the material. Conversely, a lower ratio *I*_D_/*I*_G_ implies a more regular crystal structure and higher graphitization degree of the material.^22^ The Raman spectroscopy (Fig. 3g) results show that the *I*_D_/*I*_G_ ratio of pHC is 1.59, indicating that pHC itself has a certain number of structural defects and an overall low graphitization degree. This is consistent with the typical structural characteristics of HC, namely the disordered stacking of graphite-like microcrystals. After failure treatments at different temperatures, the *I*_D_/*I*_G_ ratios of the samples exhibit differentiated variations. The *I*_D_/*I*_G_ ratios of 25 °C-HC and 60 °C-HC samples decrease slightly to 1.43 and 1.45, respectively, compared with that of the pHC. This suggests that local graphitization reconstruction may occur inside the HC materials under these two temperature conditions, where partial disordered structures are transformed into ordered sp^2^-hybridized carbon layers, resulting in a slight reduction in structural defects. In contrast, the *I*_D_/*I*_G_ ratio of the −20 °C-HC sample surges to 2.04, representing a substantial increase of 28.3% relative to pHC. This result demonstrates that the low-temperature failure process significantly exacerbates the internal structural damage of HC materials, generating a large number of new defects (*e.g.*, lattice fracture, interlayer disorder), which leads to a sharp decrease in graphitization degree and a dramatic deterioration in structural order. These findings are consistent with the phenomenon of structural damage induced by sodium dendrite growth at low temperatures observed *via* SEM in the previous section.

**Fig. 4:**
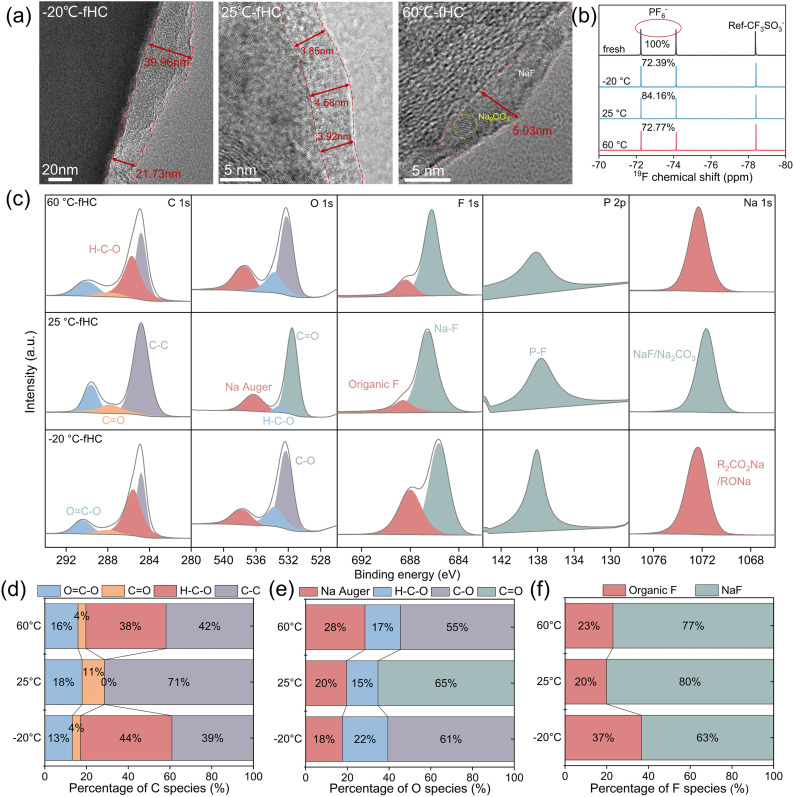
(a) HRTEM images of −20 °C-fHC, 25 °C-fHC and 60 °C-fHC. (b) ^19^F NMR spectra of electrolytes before and after cycling at −20 °C, 25 °C, and 60 °C. (c) The normalized XPS fitting spectra of C 1s, O 1s, F 1s, P 2p and Na 1s of −20 °C-fHC, 25 °C-fHC and 60 °C-fHC. (d) The proportion of different C species in the C 1s spectra, (e) the proportion of different O species in the O 1s spectra and (f) the proportion of different F species in the F 1s spectra of −20 °C-fHC, 25 °C-fHC and 60 °C-fHC.

As a core functional layer at the electrode–electrolyte interface, the SEI directly regulates Na^+^ transport kinetics, interfacial impedance, and the continuous electrolyte decomposition process, thereby dominating the cycling stability of batteries over a wide temperature range.^23,24^ In this study, high-resolution transmission electron microscopy (HRTEM) was employed to characterize the microstructure, thickness, and crystallization characteristics of the SEI layer at the atomic scale (Fig. 4a). The results demonstrate that temperature exerts a significant regulatory effect on the SEI structure: after cycling at 25 °C, a thin, continuous, and uniform SEI layer with a maximum thickness of ∼4.58 nm was formed on the surface of the HC electrode; cycling at 60 °C led to a slight increase in the maximum SEI thickness to 5 nm, accompanied by a thin but heterogeneous morphology; in contrast, cycling at −20 °C induced a pronounced SEI thickening tendency with extremely poor uniformity, where the local thickness surged to 39.96 nm, nearly 8 times higher than that at room temperature. A thin and uniform SEI can effectively block electrode–electrolyte contact, reduce interfacial impedance, and ensure rapid Na^+^ transport, which is critical for maintaining the excellent electrochemical performance of batteries.^25,26^ Conversely, a thick and heterogeneous SEI exhibits two major drawbacks: first, it extends the Na^+^ transport pathway and increases diffusion resistance, resulting in a significant rise in interfacial impedance; second, structural heterogeneity triggers local stress concentration, which causes SEI fracture during the volume expansion and contraction of the electrode upon charge–discharge cycles.^27^ The exposed fresh electrode surface further reacts with the electrolyte, initiating a “fracture-reconstruction” vicious cycle that exacerbates SEI thickening and impedance escalation, thus severely impairing the electrochemical stability of the electrode.

Notably, the SEI layer on the HC surface underwent distinct crystallization after cycling at 60 °C. Lattice fringe analysis *via* HRTEM combined with phase database matching revealed that these characteristic lattice fringes could be attributed to typical inorganic components such as Na_2_CO_3_ and NaF.^28,29^ Previous studies have confirmed that inorganic components in the SEI are pivotal to Na^+^ transport performance, as the Na^+^ transport properties mainly depend on the intrinsic ionic conductivity of inorganic species including NaF and Na_2_CO_3_, whose presence constitutes the core basis for guaranteeing the ionic transport capability of the SEI. However, it should be emphasized that the Na^+^ transport kinetics deteriorate dramatically when these inorganic components exist in a crystalline state. The highly ordered lattice arrangement of crystalline inorganic compounds strictly confines Na^+^ transport within specific lattice interstices, while the disordered atomic arrangement at grain boundaries generates substantial grain boundary resistance. These two factors synergistically elevate the Na^+^ migration energy barrier and drastically reduce the diffusion rate.^30,31^

Liquid-state ^19^F nuclear magnetic resonance spectroscopy (^19^F NMR) was employed to analyze the changes in the chemical environment of the electrolyte before and after cycle failure.^32,33^ NaCF_3_SO_3_ was introduced as an internal standard to evaluate the decomposition degree of NaPF_6_ in the electrolyte after cycling failure. The test results showed that the relative content of PF_6_^−^ decreased to 72.39% in the electrolyte of batteries failed at −20 °C, recovered to 84.16% for those failed at 25 °C, and dropped back to 72.77% for batteries failed at 60 °C, which was basically comparable to the low-temperature failure level. The results reveal that irreversible consumption of sodium salts occurs to a certain extent at all tested temperatures, with considerably higher consumption levels under low- and high-temperature conditions compared with room temperature. Such consumption can be attributed to the formation of the SEI film. Nevertheless, this degree of electrolyte decomposition is insufficient to dominate battery capacity degradation, indicating that electrolyte failure is not the core cause of full-cell failure.^34,35^

To further clarify the chemical composition characteristics of the SEI at different temperatures, X-ray photoelectron spectroscopy (XPS) was employed to systematically analyze the chemical composition of the SEI layer on the HC anode surface after 200 charge–discharge cycles (Fig. 4c).^36^ The survey scan spectra (Fig. S8) verified the presence of five characteristic elements (C, O, F, P, Na) on the surface of HC samples failed after cycling at the three temperatures. The C and O elements mainly originated from the decomposition products of electrolyte solvent molecules and the surface functional groups of the HC substrate; the F and P elements corresponded to the decomposition products of fluorinated additives and sodium salt electrolytes in the electrolyte; the presence of Na directly confirmed the existence of characteristic components related to Na^+^ conduction in the SEI layer.^37,38^ Combined with the XPS peak-fitting spectra in Fig. 4c and the quantitative analysis results in Fig. 4d–f, the SEI film formed on the HC surface at 25 °C is dominated by inorganic components such as NaF and Na_2_CO_3_. The inorganic-dominated SEI generally possesses high chemical stability and ionic conductivity, which reasonably accounts for the superior cycling stability of the cell at room temperature.^39,40^ In sharp contrast, after cycling at −20 °C and 60 °C, the relative content of organic oxygen-containing species (H–C–O/C=O) in the C 1s spectra increases remarkably (48% at −20 °C, 42% at 60 °C, and only 11% at 25 °C); meanwhile, the C–O peak dominates the O 1s spectra.^41^ These results demonstrate that the SEI layers formed at both low and high temperatures are organic-dominated. Such organic-rich SEI films usually exhibit low ionic conductivity, which directly increases the interfacial impedance of the cell.^42,43^ Notably, although the SEI formed at 60 °C is also organic-dominated, its thickness is comparable to that at room temperature. Combined with the SEI crystallization behavior observed in the previous HRTEM characterization, it can be inferred that SEI crystallization suppresses the excessive growth of the film to a certain extent, thereby alleviating the continuous thickening issue commonly existing in organic-rich SEI layers.^44,45^

## Conclusions

In summary, temperature exerts a significant regulatory effect on the electrochemical performance and failure pathways of HC anodes. At −20 °C, low temperature reduces electrolyte ionic conductivity and increases the interfacial charge transfer barrier. This causes overall kinetic sluggishness, which further induces Na dendrite growth and continuous SEI thickening. Both processes accelerate active sodium consumption and interfacial impedance accumulation, ultimately leading to irreversible capacity decay. Notably, this work differs from the conventional view that high temperatures simply cause disordered SEI thickening. Using HRTEM, we observed for the first time a crystalline SEI layer rich in nanograins on the HC surface after high-temperature cycling at 60 °C. Although this crystallization suppresses excessive SEI growth, its dense lattice structure significantly raises the Na^+^ desolvation energy barrier and interfacial migration impedance. The resulting high overpotential further drives electrolyte decomposition, forming a vicious cycle of: “aggravated side reactions → SEI crystallization → increased impedance → further side reactions”. This study systematically elucidates the failure origins and intrinsic regulation mechanisms of HC anodes under different temperature conditions, providing an important theoretical reference for the structural design and performance optimization of wide-temperature-adaptable SIBs.

## Author contributions

Rongfen Feng: experimental design, data processing, and drafting the initial manuscript. Qiang Wang: supervision. Xiaosha Wu: data curation, manuscript revision. Hanqi Zhang: data curation. Yang Gao: data curation. Ning Zhao: data curation. Jiahe Li: data curation. Xiuxia Zhao: supervision. Xiaofei Hu: writing – review and editing, supervision and project administration.

## Conflicts of interest

All authors declared that there are no conflicts of interest.

## Supplementary Material

SC-017-D6SC03211G-s001

## Data Availability

The data underlying this study are available in the published article and its supplementary information (SI), or from the authors on request. Additional data from the characterization of materials (experimental procedures for electrode preparation, battery assembly, and electrochemical performance testing; the test spectra and corresponding fitting data of EIS; the CV curves at different scan rates and the fitting results of pseudocapacitive contribution; details of theoretical calculations; the characterization results of EDS and XPS). Supplementary information is available. See DOI: https://doi.org/10.1039/d6sc03211g.
