# Supplementary material for: Mechanistic insights into interfacial failure of hard carbon anodes in sodium-ion batteries under extreme conditions
Source: Chem Sci. 2026 May 21;17(27):13423–32. doi: 10.1039/d6sc03211g (PMC13224049; doi:10.1039/d6sc03211g)
Supplement: SC-017-D6SC03211G-s001 [file SC-017-D6SC03211G-s001.pdf]

Supplementary Information (SI) for Chemical Science.
This journal is © The Royal Society of Chemistry 2026

Supporting Information

Mechanistic insights into interfacial failure of hard carbon anodes in sodium-ion batteries under extreme conditions

Rongfen Feng,[ab] Qiang Wang,[c] Xiaosha Wu,[a] Hanqi Zhang,[a] Yang Gao,[a] Ning Zhao,[a] Jiahe Li,[a] Xiuxia Zhao,[a] Xiaofei Hu*[ab]

a. *School of Chemistry, Xi'an Jiaotong University, Xi'an 710049, Shaanxi, China*

b. *National Innovation Platform (Center) for Industry-Education Integration of Energy Storage Technology, Xi'an Jiaotong University, Xi'an 710049, Shaanxi, China. E-mail: xiaofei.hu@xjtu.edu.cn*

c. *CATARC Intelligent Technology (Tianjin) Co., Ltd., Tianjin, 300300, China*

Experimental Procedures

Materials

Both commercial sodium vanadium phosphate ($Na_3V_2(PO_4)_3$, NVP) and hard carbon (HC) were directly employed without any further modification or pretreatment. The cathode slurry was prepared by dispersing NVP, conductive carbon black (Super P) and polyvinylidene fluoride (PVDF) at a weight ratio of 8:1:1 in N-methyl-2-pyrrolidone (NMP) solvent. Subsequently, the resultant slurry was uniformly cast onto an aluminum (Al) current collector using a doctor blade. The as-coated films were dried in a vacuum oven at 100 °C for 12 h, followed by punching into circular disks with a diameter of 12 mm. The average active material loading was measured to be 2.43 mg cm^{-2}. Fabrication of the anode electrode: For the anode electrode, the slurry was formulated by mixing Kuraray HC, Super P, and PVDF at a weight ratio of 8:1:1 in NMP solvent. The slurry was then blade-coated onto a copper (Cu) current collector. After drying in a vacuum oven at 100 °C for 12 h, the films were punched into circular disks with a diameter of 14 mm, yielding an average active material loading of 1.22 mg cm^{-2}.

Electrochemical measurements

The assembly of coin cells (CR2032) was conducted in an argon-filled glove box. The experiments involved the assembly and testing of three categories of cells, namely pristine HC (pHC) half-cells, NVP //HC full-cells, and Post-cycling-failure HC (fHC) half-cells. The specific configurations and testing protocols are detailed as follows: Cell assembly configurations: HC half-cells: pHC was employed as the working electrode, metallic Na foil as the counter electrode, 1.0 M $NaPF_6$ in diglyme as the electrolyte, and glass fiber filter paper (GF/D) as the separator. NVP//HC full-cells: The full-cells were assembled

with NVP as the cathode and presodiated HC as the anode, coupled with the same electrolyte and separator as those used in HC half-cells. The N/P ratio was set to 1.2. fHC half-cells: The fHC were harvested by disassembling the cycled-degraded NVP//HC full-cells, and then reassembled with metallic Na foil to fabricate fHC half-cells. High-low temperature long-term cycling Test: After the assembly of coin cells, the cells were first rested at room temperature for 8 h, then transferred into a high-low temperature test chamber and kept isothermally for 1 h before initiating the long-cycle test. An independent thermometer was placed throughout the entire test process for real-time temperature monitoring to ensure consistency between the displayed temperature of the chamber and the actual ambient temperature around the cells. Electrochemical test platforms: Galvanostatic charge-discharge tests (GCD) and galvanostatic intermittent titration technique (GITT) measurements were implemented on a Neware battery test system. The ionic conductivity, electrochemical impedance spectroscopy (EIS) and cyclic voltammetry (CV) tests were carried out using an electrochemical workstation (CHI6620E). Specific test procedures and parameters: Presodiation of HC: The as-assembled HC half-cells were subjected to electrochemical presodiation. The test was performed within a voltage window of 0.01-1.5 V at a current density of 20 mA g^{-1}, following a discharge-charge protocol for 3.5 cycles. Long-term cycling of full-cells: The presodiated HC was paired with NVP to assemble the full-cells, which were then subjected to long-term cycling tests under different temperature conditions. The cycling tests were conducted in a voltage range of 2.0-3.8 V at a high current density of 5 C, where the C-rate was defined as 1 C = 117 mA g^{-1}. GITT measurements: GITT tests were performed on both pHC half-cells and fHC half-cells within the identical voltage window of 0.01-1.5 V. The testing parameters were set as

follows: a charge-discharge current density of 20 mA g^{-1}, a single charge-discharge duration of 30 min, and a relaxation period of 90 min. Based on the GITT profiles, the sodium-ion diffusion coefficient (D_{Na+}) was calculated using the following equation:

$$D_{Na^+} = \frac{4}{\pi\tau}\left(\frac{m_B V_M}{M_B S}\right)\left(\frac{\Delta E_S}{\Delta E_\tau}\right)^2$$

where M_B and V_M represent the molar weight and volume of the cathode material, m_B is the molecular weight, S is the surface area of the electrode, and τ is the relaxation time. ΔE_S and ΔE_τ are the steady voltage change and total variation of battery voltage during the current pulse, respectively. EIS and CV characterizations were performed on pHC half-cells and fHC half-cells. For EIS tests, the frequency range was set from 0.001 Hz to 10^5 Hz. CV tests were conducted over a voltage range of 0.01-2.0 V at various scan rates. The ionic conductivity was evaluated in stainless-steel symmetrical batterie and calculated based on the equation of $\sigma = L/(SR)$ (σ represents the ionic conductivity, R represents the intercept of the Nyquist plot and Z' axis, L and S are the distance and area of stainless-steel).

Material Characterization

The scanning electron microscopy (SEM) (GeminiSEM 360, ZEISS, Germany) coupled with energy-dispersive X-ray spectroscopy (EDS) was employed to conduct synchronous characterization of the electrode materials in terms of their micromorphology, structural characteristics, and elemental distribution. X-ray diffraction (XRD) (D8 ADVANCE, Bruker, Germany) technique was utilized to determine the phase composition and crystal structure of the samples. X-ray photoelectron spectroscopy (XPS) (K-Alpha, Thermo Fisher, USA) measurements were performed to conduct accurate analysis of the elemental composition and electronic valence states on the sample surface. To eliminate

instrumental errors, all binding energy values were calibrated using the C 1s peak at 284.8 eV as the internal standard. Furthermore, high-resolution transmission electron microscopy (HRTEM) (Tecnai G2 F30, Thermo Fisher, USA) was adopted to characterize the microstructure, thickness and morphological characteristics of the solid electrolyte interphase (SEI) film formed on the surface of the failed hard carbon electrode. In addition, Raman (inVia Reflex, RENISHAW, UK) spectroscopy was applied to analyze the carbon structural order, defect density and chemical bond vibration characteristics of the samples, and the structural disorder degree of the samples was quantified by the intensity ratio of the D band to the G band (I_D/I_G). Liquid-state nuclear magnetic resonance (^{19}F NMR) (AVANCE NEO 400, Bruker, Germany) spectroscopy was utilized to perform quantitative analysis of the electrolyte within the battery system both before and after cycling.

Theoretical Calculations

All periodic DFT calculations for periodic material systems were performed with the Vienna Ab initio simulation package (VASP) using the projector-augmented wave (PAW) method. The exchange–correlation function was handled using the generalized gradient approximation (GGA) formulated by the Perdew-Burke-Ernzerhof (PBE). The van der Waals (vdW) interactions are described with the DFT-D3 method in Grimme's scheme. The interaction between the atomic core and electrons was described by the projector augmented wave method. The plane-wave basis set energy cutoff was set to 500 eV. The Brillouin zone was sampled with a 2×2×1 grid centered at the gamma (Γ) point for geometry relaxation. All the slabbed models possessed a vacuum spacing of ≈ 15 Å sampled, ensuring negligible lateral interaction of adsorbates. The bottom layers about half of the structure were kept frozen at the lattice position. All structures were fully relaxed to

optimize without any restriction until their total energies were converged to < 1×10^{-6} eV, and the average residual forces were < 0.02 eV/Å.

All molecular quantum chemical calculations were performed using the ORCA program package. Geometry optimization of the $NaPF_6$ ion pair was carried out at the B3LYP level of theory incorporating Grimme's D3 dispersion correction (B3LYP-D3). The def2-TZVP(-f) basis set was employed for all atoms. To enhance computational efficiency, the resolution of identity (RI) approximation for the Coulomb term and the chain-of-spheres (COSX) approximation for the exchange term were applied, using the def2/J auxiliary basis set. The optimized structure was then used for subsequent harmonic frequency calculations at the same theoretical level to obtain thermodynamic corrections. The frequency calculations were performed at two distinct temperatures, 298.15 K and 253.15 K, to simulate ambient (25 °C) and low-temperature (-20 °C) conditions, respectively. The Gibbs free energy of dissociation (ΔG_{diss}) was derived from the difference in Gibbs free energies between the fully relaxed separated ions (Na^+ + PF_6^-) and the $NaPF_6$ ion pair, including zero-point energy and thermal contributions.

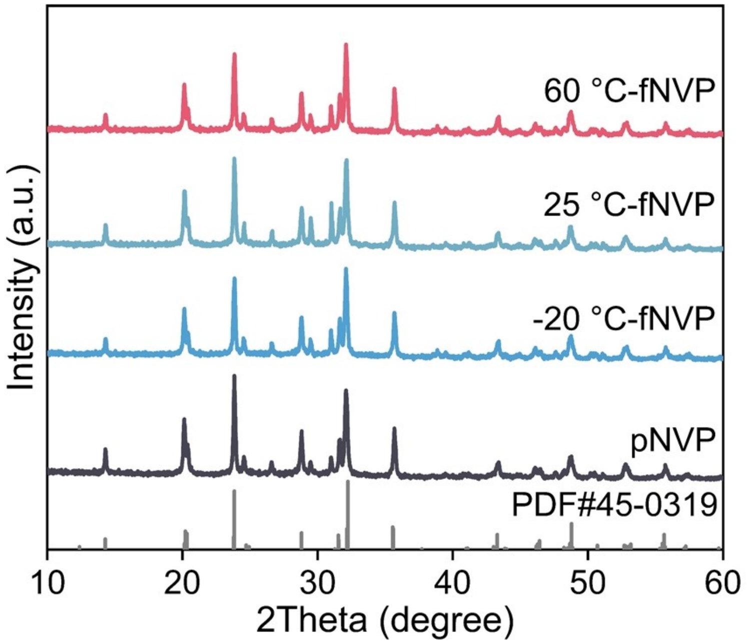

Fig. S1. XRD patterns of pristine NVP electrode and NVP electrodes failed after cycling at different temperatures.

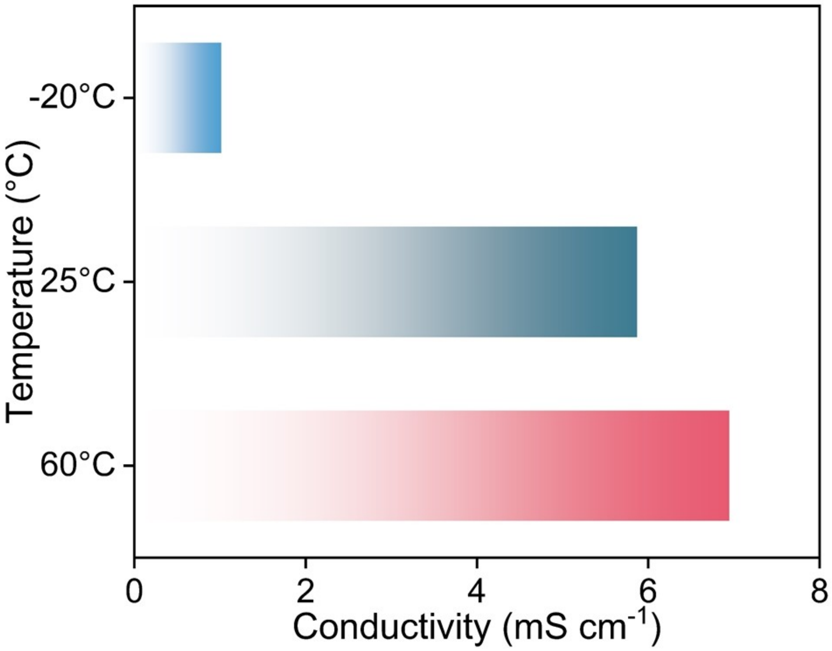

Fig. S2. Ionic conductivity of electrolytes at different temperatures.

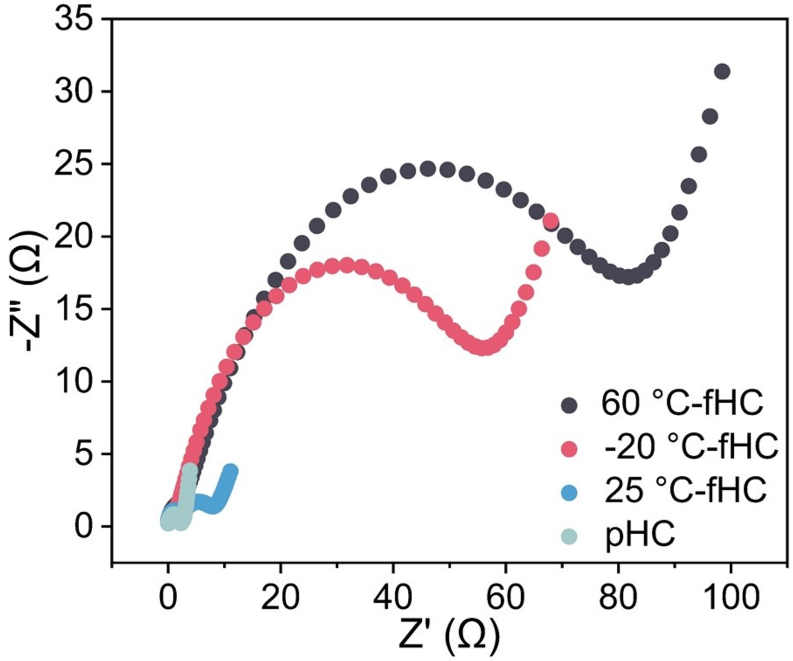

Fig. S3. Nyquist plots of pHC//Na batteries, -20 °C-fHC//Na batteries, 25 °C-fHC//Na batteries and 60 °C-fHC//Na batteries at 25 °C.

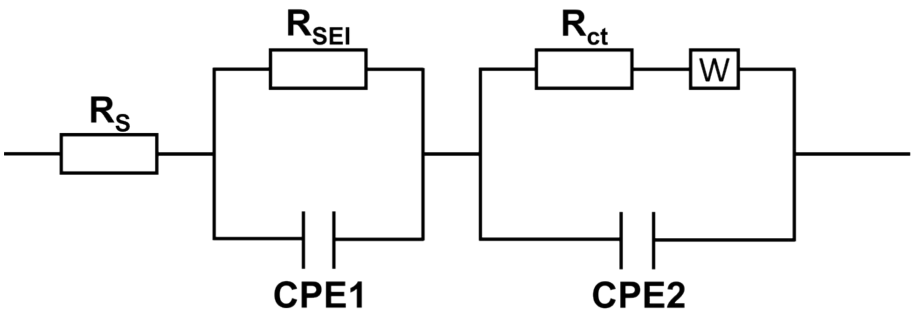

Fig. S4. The equivalent circuit of the impedance spectra.

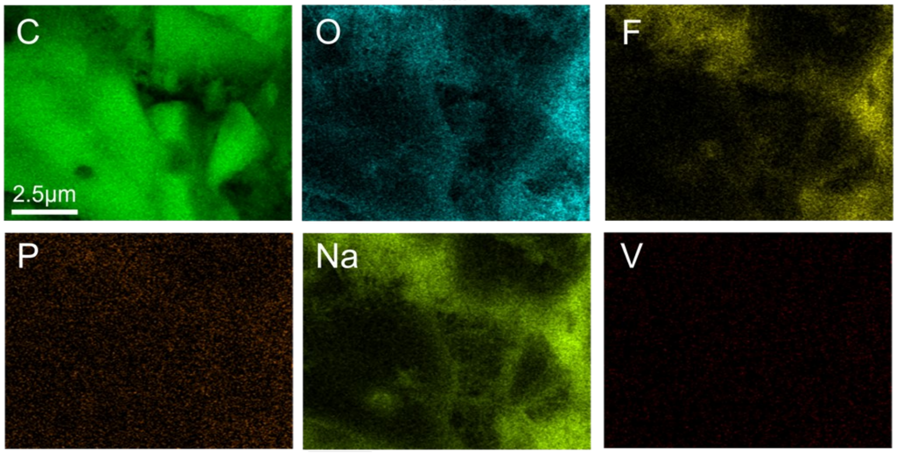

Fig. S5. EDS mapping of HC after cycling failure at -20 °C.

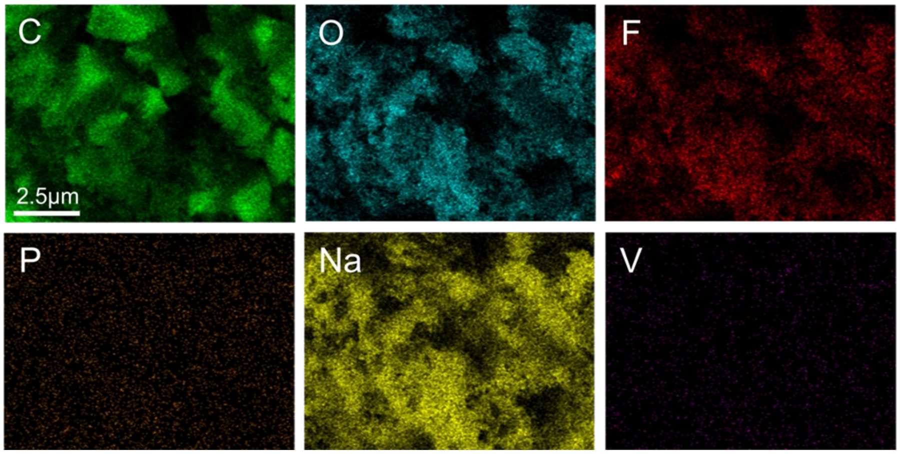

Fig. S6. EDS mapping of HC after cycling failure at 25 °C.

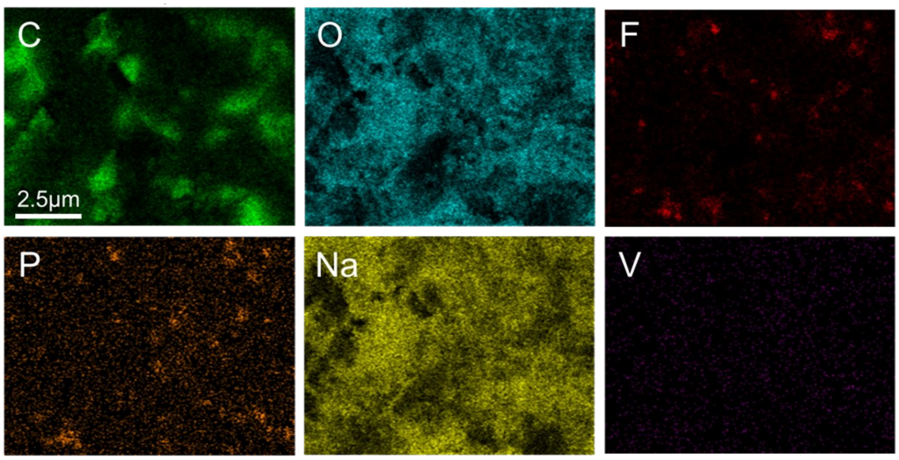

Fig. S7. EDS mapping of HC after cycling failure at 60 °C.

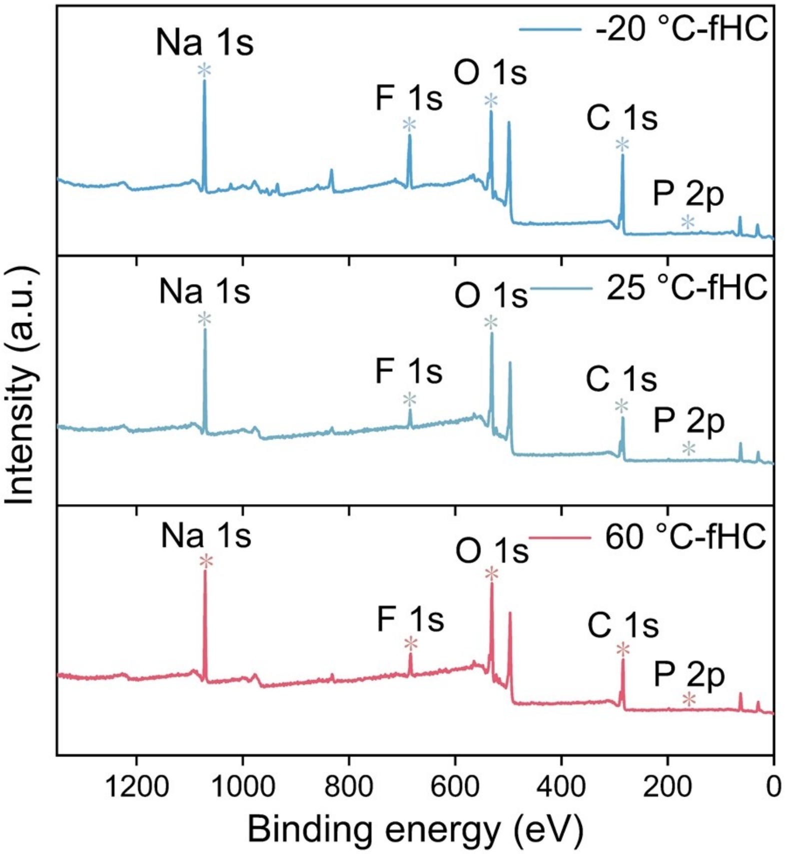

Fig. S8. XPS spectra of HC after cycling at -20 °C, 25 °C, and 60 °C.

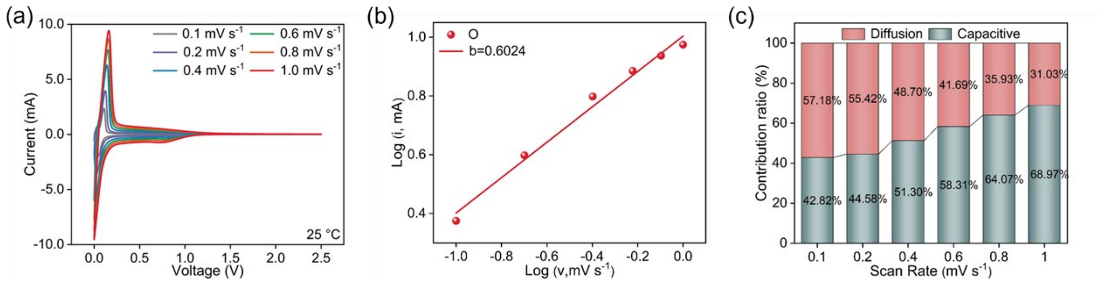

Fig. S9. (a) Cyclic voltammetry profiles of HC//Na half-cells measured at 25 °C with various scan rates; (b) Linear fitting curves between peak current and scan rate; (c) Quantitative plots of pseudocapacitive contribution ratio.

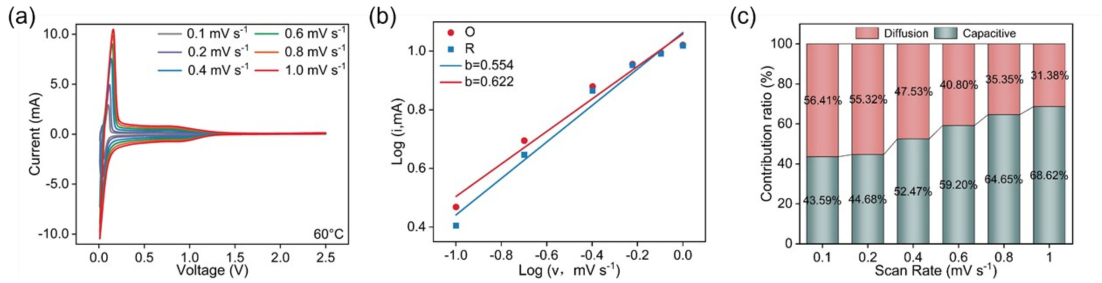

Fig. S10. (a) Cyclic voltammetry profiles of HC//Na half-cells measured at 60 °C with various scan rates; (b) Linear fitting curves between peak current and scan rate; (c) Quantitative plots of pseudocapacitive contribution ratio.

Table S1. The fitting results of the equivalent circuit of pHC //Na batteries at different temperatures.

Temperature	R_s		R_{ct}	
	**Value(Ω)**	**Error(%)**	**Value(Ω)**	**Error(%)**
-10 ℃	11.36	0.51	4.91	10.52
0 ℃	6.26	0.87	3.22	3.13
25 ℃	5.40	0.42	2.13	1.55
40 ℃	4.78	0.47	1.38	2.48

Table S2. The fitting results of the equivalent circuit of -20 ℃-fHC //Na batteries at different temperatures.

Temperature	R_s		R_{ct}	
	Value(Ω)	Error(%)	Value(Ω)	Error(%)
-10 ℃	11.09	2.19	1511.02	0.94
0 ℃	7.49	1.73	445.03	5.51
25 ℃	5.26	0.96	60.69	1.50
40 ℃	4.48	0.52	25.20	1.06

Table S3. The fitting results of the equivalent circuit of 25 ℃-fHC //Na batteries at different temperatures.

Temperature	R_s		R_{ct}	
	**Value(Ω)**	**Error(%)**	**Value(Ω)**	**Error(%)**
-10 ℃	6.04	1.76	45.58	2.62
0 ℃	8.20	0.91	31.05	2.49
25 ℃	5.16	0.88	6.84	5.41
40 ℃	4.54	0.69	2.69	11.46

Table S4. The fitting results of the equivalent circuit of 60 ℃-fHC //Na batteries at different temperatures.

Temperature	R_s		R_{ct}	
	**Value(Ω)**	**Error(%)**	**Value(Ω)**	**Error(%)**
-10 ℃	29.03	1.94	3093.01	1.09
0 ℃	10.90	1.94	837.70	3.13
25 ℃	7.16	0.74	90.01	0.87
40 ℃	8.20	0.69	35.52	1.61

Table S5. The fitting results of the equivalent circuit of pHC//Na batteries, -20 ℃-fHC//Na batteries, 25 ℃-fHC//Na batteries and 60 ℃-fHC//Na batteries at 25 ℃.

	**R_s**		**Rsei**		**R_{ct}**	
	**Value(Ω)**	**Error(%)**	**Value(Ω)**	**Error(%)**	**Value(Ω)**	**Error(%)**
pHC//Na	5.26	0.35	-	-	1.61	1.55
-20 ℃-fHC//Na	5.26	0.96	1.19	9.48	60.69	1.50
25 ℃-fHC//Na	5.16	0.88	2.01	8.30	6.84	5.41
60 ℃-f HC//Na	7.16	0.74	2.12	5.77	90.01	0.87

Table S6. Weight percentages (%) of elements from EDS mapping for HC before and after cycling at -20 °C, 25 °C, and 60 °C.

Element	pHC	-20 °C-fHC	25 °C-fHC	60 °C-fHC
C	97.46	83.18	57.01	37.96
O	0.68	7.82	18.57	33.05
F	1.81	3.15	5.26	2.89
Na	0.00	5.79	18.85	25.08
P	0.03	0.05	0.09	0.66
V	0.02	0.00	0.23	0.36
